# Mucosal microRNAs relate to age and severity of disease in ulcerative colitis

**DOI:** 10.18632/aging.202715

**Published:** 2021-03-01

**Authors:** Mikkel Malham, Jaslin P. James, Christian Jakobsen, Estrid Hoegdall, Kim Holmstroem, Vibeke Wewer, Boye S. Nielsen, Lene B. Riis

**Affiliations:** 1 The Pediatric Department, Copenhagen University Hospital, Hvidovre 2650, Denmark; 2 The Pediatric Department, Holbaek Hospital, Holbaek 4300, Denmark; 3 Department of Pathology, Copenhagen University Hospital, Herlev 2730, Denmark; 4 Biomedical Technology, Bioneer A/S, Hoersholm 2970, Denmark

**Keywords:** pediatric, inflammatory bowel disease, quantitative polymerase chain reaction, miRNA, in situ hybridization

## Abstract

Despite significant evidence that the expression of several microRNAs (miRNAs) impacts disease activity in patients with ulcerative colitis (UC), it remains unknown if the more severe disease phenotype seen in pediatric onset UC can be explained by an altered miRNA expression. In this study, we assessed the relationship between miRNA expression, age, and disease severity in pediatric and adult patients with UC. Using RT-qPCR, we analyzed the expression of miR-21, miR-31, miR-126, miR-142 and miR-155 in paraffin embedded rectum biopsies from 30 pediatric and 30 adult-onset UC patients. We found that lesions from adult patients had significantly higher expression levels of miR-21 compared to pediatric patients and that the expression levels of miR-31 (all patients) and miR-155 (pediatric patients only) correlated inversely with histological assessed disease severity. Using *in situ* hybridization followed by image analysis, the expression level estimates of miR-21 and miR-126 correlated with histological assessed disease severity. In conclusion, we found that the expression of miRNAs depends on the age of the patient and/or the severity of the disease, suggesting that miRNAs may contribute to the regulation of inflammation in UC and could be useful biomarkers in the surveillance of disease severity.

## INTRODUCTION

Ulcerative colitis (UC) is an inflammatory bowel disease (IBD) characterized by a chronic inflammation of the colon. The extent of the disease varies, but adult-onset UC (aUC) typically presents with a distal proctitis (limited disease extent). By contrast pediatric onset UC (pUC) typically presents with the entire colon affected (extensive disease extent) [1]. From these observations, it has been hypothesized that pUC represents a more severe disease phenotype which is reflected by a more frequent use of systemic steroids, thiopurines, and biologics in patients with pUC [2, 3].

Since the etiology of UC is poorly understood, it is unknown whether the difference in phenotype between pUC and aUC is caused by a fundamental difference in disease pathogenesis. It is thought that UC develops due to an aberrant immune response towards the intestinal microbiota in genetically disposed individuals [4]. Interestingly, microRNAs (miRNA), a group of short non-coding RNA regulating mRNA translation [5], are differentially expressed in UC compared to healthy controls and are therefore thought to be involved in UC pathogenesis [6–9].

In aUC, the expression of several miRNAs is altered in colon mucosa [10–19] and blood [13, 20] compared to both healthy controls and patients with Crohn’s disease.

MiRNA-21 is likely the most extensively described miRNA in UC [21]. In a rodent colitis model using Il-10 knockout mice, miRNA-21 expression was significantly increased in the colon tissue compared to wildtype mice [21]. In the same study, when inducing colitis with the dextran sulphate sodium (DSS) method, the authors found an increased survival rate in miR-21 knockout mice compared to wildtype mice as well as significantly decreased levels of tumor necrosis factor alpha (TNF-α), a proinflammatory cytokine, which indicates that miR-21 stimulates the inflammatory process. Additionally, miR-21 is downregulated in UC patients in remission leading to the speculation of targeting miR-21 as a novel treatment strategy [22]. MiR-21 has also been found to be a diagnostic marker that can help to differentiate Crohn’s disease (CD) from UC, which otherwise can be a diagnostic challenge with up to nine percent being diagnosed with unclassified IBD [20, 23]. Schaefer et al*.* reported a panel of six miRNAs (including miR-21) to differentiate CD from UC [13], and using quantitative *in situ* hybridization (qISH), Thorlacius-Ussing et al*.* [10] found significantly higher levels of miR-21 in tissue from UC patients compared to that from CD patients.

Several studies have reported on the role of other miRNAs in IBD in general, both exploring mechanistic roles and the potential role of miRNA as biomarkers and drug targets [4, 6, 24]. In particular, miR-155 has been linked to IBD and to inflammation in a variety of other inflammatory diseases, including psoriasis and atherosclerosis [25, 26]. In a study using the DSS-induced colitis model, loss of miR-155 was found to ameliorate colitis by reducing TNF-α and interleukin- 6, -12 and -17 in the tissue [27]. In another DSS study, miR-155 was associated with Th17 cell maturation which caused increased levels of interleukin-23 [28]. Yet, while several studies support a pro-inflammatory effect of miR-155 [29], one study found miR-155 to have a regulatory effect by targeting the expression of interleukin-1 in human dendritic cells [30].

Based on the fact that the existing literature consistently reports differential miRNA expression in UC patients compared to healthy controls [10, 13, 14, 31], we selected the following five miRNAs for our study: miR-21-5p, miR-31-5p, miR-126-3p, miR-142-5p and miR-155-5p. We compared the expression of the five miRNAs in pUC and aUC and evaluated their performance in assessing the grade of inflammation. As previously mentioned, miR-21 and miR-155 are involved the inflammatory process of IBD. MiR-126 is expressed in endothelial cells and represents the degree of vascularization of the tissue [32, 33]. MiR-142 is expressed primarily in T cells and represent the prevalence of immune cells [34]. The cellular origin of miR-31 in inflammatory tissue is not yet resolved. Here we performed ISH for miR-21 and miR-126, for which we had well-performing ISH assays, and correlated the expression levels with the clinical and histological parameters.

## RESULTS

We included 60 patients equally distributed among the following four groups: *de novo* diagnosed pediatric patients (new pUC), previously diagnosed pediatric patients (known pUC), *de-novo* diagnosed adult patients (new aUC), and previously diagnosed adult patients (known aUC).

Baseline characteristics are presented in Table 1. Endoscopic remission was observed in five patients while histological assessed remission (Geboes score <2) was recorded in 20 patients (Table 1). No differences in disease extent or activity were found between the pediatric and adult patients. miRNA expression level estimates are presented in Table 2.

**Table 1:** Patient characteristics, laboratory values and medical treatment.

	**Pediatric patients**			**Adult patients**	
	**De novo**	**Previously diagnosed**		**De novo**	**Previously diagnosed**
**Demographics**					
Patients (n)	15	15		15	15
Sex (n)					
Male	6	5		7	6
Female	9	10		8	9
Median age (IQR)	13 (9-15)	15 (11-16)		29 (25-40)	31 (25-39)
**Disease parameters**					
Phenotype (n)					
Remission	-	3		-	2
Proctitis	5	4		6	2
Left sided	1	3		4	2
Extensive	9	5		5	9
UCEIS (median (IQR))	3 (1-6)	1 (0-4)		3 (1-5)	1 (0-4)
Geboes score (n)					
0-1 / 2-3 / >3	2 / 9 / 4	6 / 6 / 3		4 / 3 / 8	8 / 5 / 2
**Biomarkers (median [IQR])**					
Fecal calprotectin (μg/g)^a^	1340 (97-1800)	525 (62-1080)		1800 (1130-1800)	1042 (300-1800)
C-reactive protein (mg/L)	1 (0-9)	1 (0-2)		2 (0-5)	8 (3-22)
Albumin (g/L)	37 (34-40)	38 (36-39)		38 (35-43)	36 (33-40)
Hemoglobin (mmol/L)	7.5 (7.1-8.3)	7.8 (6.9-8.1)		8.6 (8.1-9.5)	8.1 (7.5-8.8)
Erythrocyte sedimentation rate	10.5 (3-16)	6 (4-18)		5 (3-16)	13 (3-21)
Neutrophils (E^9/L)	4 (3-6)	4 (3-4)		4 (4-5)	6 (4-8)
**Treatment (n)**					
Oral 5-aminosalicylic acid	-	12		4	10
Topic 5-aminosalicylic acid	-	3		3	9
Systemic corticosteroids	-	2		-	2
Topical corticosteroids	-	1		-	1
Thiopurines	-	7		-	4
Biologics	-	6		-	1

Due to laboratory restrictions fecal calprotectin was capped at 1800 μg/g. IQR, Interquartile range. N, number. UCEIS, Ulcerative Colitis Endoscopic Index of Severity.

**Table 2:** MicroRNA expression levels analyzed by A: reverse transcription quantitative polymerase chain reaction (qPCR) and B: quantitative image analysis of *in situ* hybridization (qISH). The qPCR values are listed as expression levels normalized to miR-103.

		**Pediatric patients**			**Adult patients**		**Correlation to UCEIS (Rho)**	**Correlation to geboes (Rho)**
		**De novo**	**Previously diagnosed**		**De novo**	**Previously diagnosed**		
qPCR (median [IQR])								
	MiRNA-21^a,b^	32.5 (20-39)	7.5 (6-12)		81.5 (58-150)	21.5 (8-36)	NS	NS
	MiRNA-31^b^	0.2 (0.1-1.0)	1.3 (0.6-6.1)		1.1 (0.3-1.5)	7.5 (0.5-18.6)	NS	NS
	MiRNA-126	1.3 (1.1-2.1)	0.2 (0.1-0.3)		0.3 (0.1-0.9	3.7 (2.3-4.9)	NS	NS
	MiRNA-142	0.5 (0.3-0.8)	0.6 (0.3-0.8)		0.7 (0.2-1.9)	0.9 (0.5-2.1)	NS	NS
	MiRNA-155	0.3 (0.2-0.6)	0.3 (0.1-2.6)		0.3 (0.1-1.1)	1.5 (0.7-2.0)	NS	NS
qISH (median [IQR])								
	MiRNA-21	0.032 (0.006-0.08)	0.012 (0.008-0.02)		0.018 (0.004-0.04)	0.028 (0.006-0.08)	0.46	0.47
	MiRNA-126	0.039 (0.02-0.07)	0.019 (0.02-0.03)		0.033 (0.02-0.05)	0.04 (0.02-0.06)	0.47	0.54

^a^Significant difference between pediatric and adult patients (p<0.05). ^b^Significant difference between de-novo and previously diagnosed patients (p<0.05). MiR, microRNA. IQR, Interquartile range. UCEIS, Ulcerative Colitis Endoscopic Index of Severity. NS, nonsignificant. The qISH values are listed as proportion of the tissue stained with the microRNA probe.

### RT-qPCR analysis of miR-21

**Figure 1:**
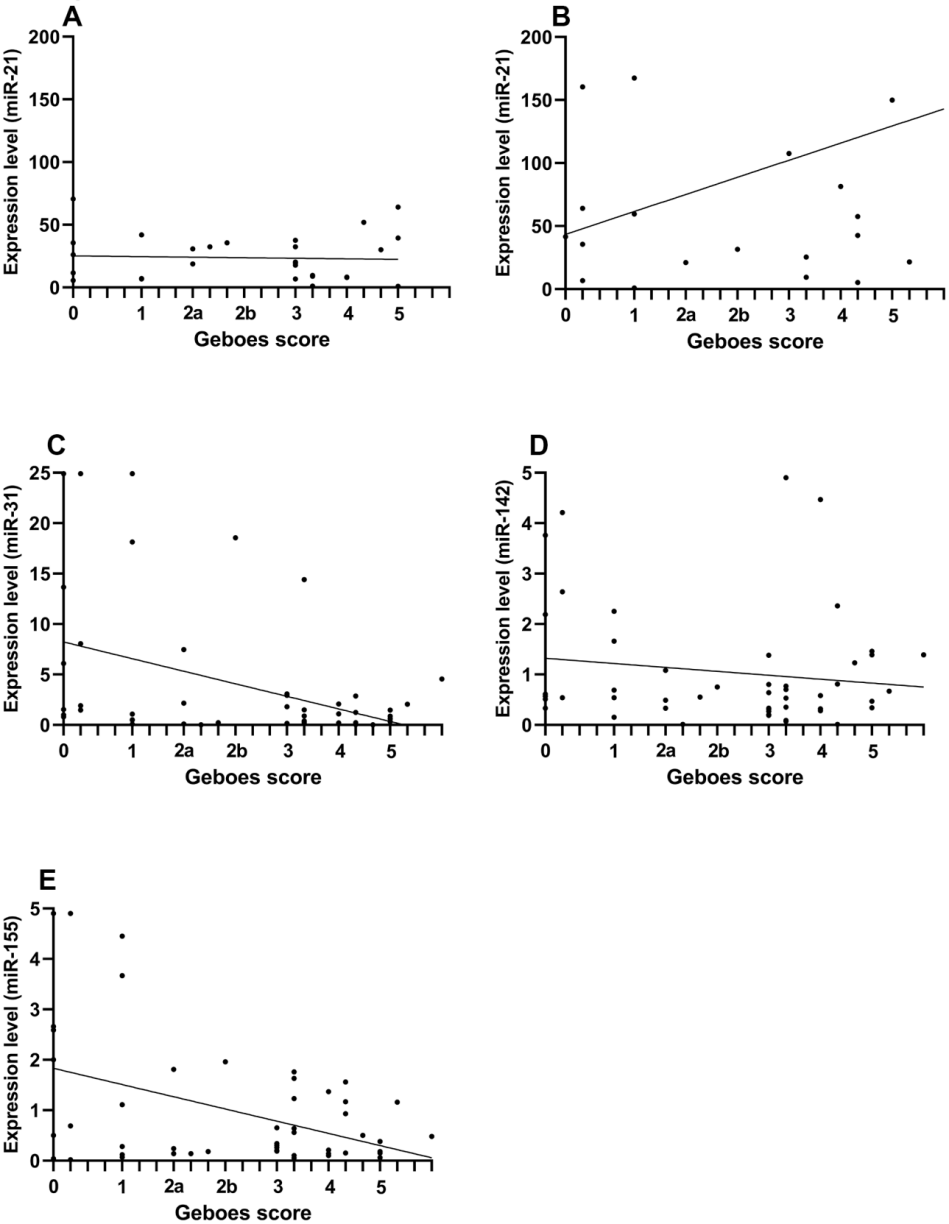
**Comparison of miRNA expression levels and Geboes score in pediatric and adult patients with ulcerative colitis.** The correlation is presented by Spearman’s rho and compares expression levels of miRNA (assessed by RT-qPCR) and Geboes score from the rectal mucosa. Because the miR-21 expression was increased in adult patient, pediatric and adult patients are presented separately for the comparison of miR-21. (**A**) pediatric miR-21 (rho=-0.04, p=0.8). (**B**) adult miR-21 (rho=0.19, p=0.37). (**C**) miR-31 (rho=-0.29, p=0.03). (**D**) miR-142 (rho=-0.04, p=0.8). (**E**) miR-155 (rho=-0.21, p=0.15).

The expression levels of miR-21 in pUC patients were significantly lower than in aUC patients (relative median fold difference [IQR]: 19 [8-36] and 50 [21-129] in pUC and aUC, respectively, p=0.003). No difference was found between patients in histological assessed remission compared to those with active disease for both pUC and aUC study groups. Among the aUC, males had higher expression levels than females (relative median fold difference [IQR]: 150 [43-203] vs 32 [21-58], p=0.02). No correlation was found between miR-21 measured by RT-qPCR and Geboes score (Figure 1).

### QISH analysis of miR-21

**Figure 2:**
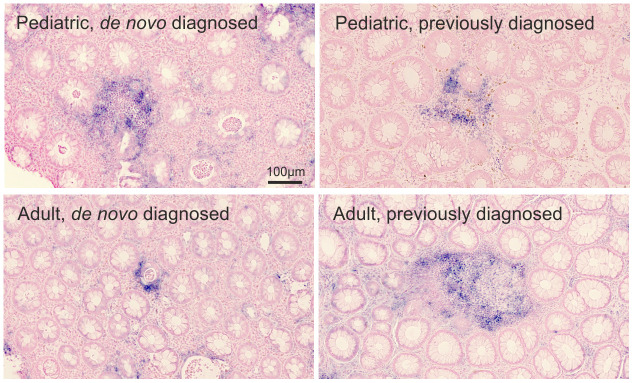
**miR-21 ISH.** Visual assessment of miR-21 expression (blue stain) in the rectal mucosa of 4 representative pediatric and adult UC patients. The miR-21 ISH signal is observed in relation to crypt abscesses. The miRNA expression by proportion of the region of interest: pediatric de novo diagnosed=0.079; pediatric previously diagnosed =0.023; adult de novo diagnosed=0.016; adult previously diagnosed=0.032. Sections were counterstained with nuclear fast red.

By ISH, we found a focal upregulation of miR-21 in association with inflamed crypts with similar expression patterns in all study groups (Figure 2).

The qISH expression level estimates (the fraction of the ROI with miRNA expression, Supplementary Figure 1) of miR-21 correlated positively with endoscopic assessed severity of disease (UCEIS) (Table 2, p=0.002), and patients in endoscopic remission had lower expression levels of miR-21 than patients with active disease (median expression level estimate [IQR]: 0.009 [0.004-0.02] vs. 0.04 [0.01-0.08], p=0.02). This association was also found when looking at miR-21 in patients in histological remission, who had lower levels than the patients with histologically active disease (median expression level estimate [IQR]: 0.006 [0.00-0.01] vs. 0.03 [0.01-0.09], p<0.0001). Furthermore, miR-21 correlated with Geboes score (Table 2, p=0.001). When stratifying by *de novo* and previously diagnosed patients, the correlation between miR-21 expression and Geboes score was higher in previously diagnosed patients (rho= 0.8, p=0.002). There was no significant correlation between the miR-21 qISH expression level estimates and the RT-qPCR based expression levels (rho=-0.02, p=0.9. Supplementary Figure 2).

### RT-qPCR analysis of miR-31

We did not see a difference in the miR-31 expression levels comparing pUC vs aUC. In comparing patients in histological remission (Geboes score <2) with patients with histological activity, we found that pUC patients in remission had increased expression levels compared to pUC patients with active disease (relative median fold difference [IQR]: 3.6 [0.9-15.9] vs. 0.2 [0.1-1.5], p=0.004). MiR-31 negatively correlated with Geboes score (rho: -0.29, p=0.03). When stratifying by time of diagnosis, miR-31 correlated with Geboes score in previously diagnosed patients (rho=-0.55, p=0.004).

### RT-qPCR analysis of miR-126

When comparing expression levels in pUC with aUC, and the patients with active disease with remission, we found no differences in the expression levels by RT-qPCR analysis.

### QISH analysis of miR-126

**Figure 3:**
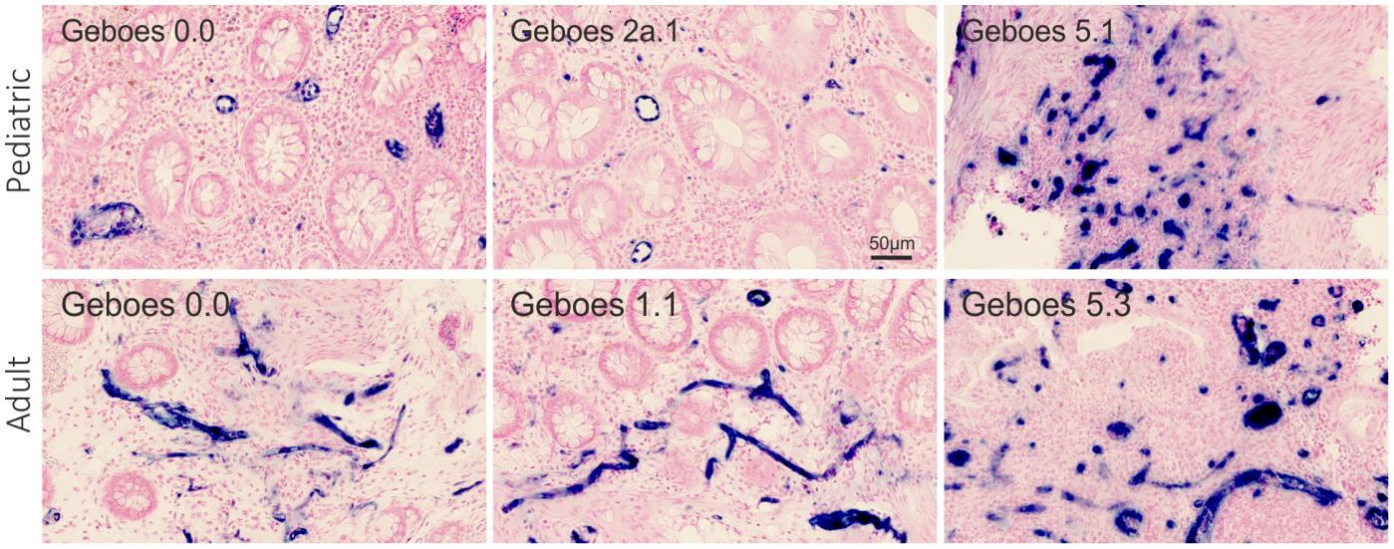
**miR-126 ISH.** Visual assessment of miR-126 expression (blue stain) in the rectal mucosa of 6 representative pediatric and adult UC patients with different Geboes scores. The miR-126 ISH signal is observed in relation to the vascular endothelial cells. The microRNA expression by proportion of the region of interest: top row from the left - 0.036, 0.024, and 0.22. Bottom row from the left - 0.025, 0.026, and 0.11.

By ISH, miR-126 was associated with the vascular endothelial cells (Figure 3) with similar expression patterns in all study groups.

The qISH expression level estimates of miR-126 positively correlated with endoscopic assessed severity of disease (UCEIS) (Table 2, p=0.002). Furthermore, miR-126 correlated with Geboes score (Table 2, p=0.0002). Examples of the varying miR-126 staining and the relation to the Geboes categories are presented in Figure 3. When stratifying by *de novo* and previously diagnosed patients, the correlation increased in previously diagnosed patients (rho= 0.92, p<0.0001). The qISH expression estimates were decreased in patients in histological remission compared to patients with active disease (median expression level estimates [IQR]: 0.02 [0.01-0.04] vs. 0.04 [0.02-0.07], p=0.01). The miR-126 expression estimates did not correlate with the RT-qPCR results (rho=0.22, p=0.2. Supplementary Figure 2).

### RT-qPCR analysis of miR-142

There was no difference in the miR-142 expression levels comparing pUC vs aUC. PUC in histological remission had significantly increased expression levels compared to pUC with histological activity (relative median fold difference [IQR]: 0.65 [0.55-2.22] vs. 0.47 [0.31-0.70], p=0.02). In previously diagnosed UC patients, miR-142 levels correlated negatively with Geboes score (rho= -0.51, p=0.01).

### RT-qPCR analysis of MiR-155

There was no difference in the miR-155 expression levels comparing pUC vs aUC. The expression levels of miR-155 were significantly increased in pUC in histological remission (relative median fold difference [IQR]: 2.3 [0.8-3.6] and 0.2 [0.1-0.3] in remission and with activity, respectively, p=0.002). In pUC, miR-155 negatively correlated with Geboes score (rho= -0.5, p=0.005). When stratifying all patients by *de novo* diagnosed and previously diagnosed, miR-155 expression levels negatively correlated with the Geboes score in previous diagnosed patients only (rho= -0.45, p=0.03).

## DISCUSSION

For unknown reasons, pUC presents with a more extensive disease than aUC and requires a more aggressive treatment regimen to induce and maintain remission [1–3]. Because the existing literature on miRNA in IBD indicates a role of miRNA in disease activity, phenotype, and susceptibility to treatment regimens [35–37], it is critical to pursue translational miRNA studies focusing on differences in expression profiles between pUC and aUC. We report that the expression of the five studied miRNAs vary according to age of the patient, chronicity of disease or severity of inflammation. MiR-21 and miR-126 were positively correlated with histological assessed disease severity, whereas miR-31, miR-142 and miR-155 were negatively correlated. Our understanding is that this is the first study that compares miRNA expression levels in pUC with those in aUC patients and correlates miRNA expression with histologically and endoscopically assessed severity of disease. Our findings suggest that the various miRNAs are involved in different inflammatory pathways of the disease, that the use of methodological application can provide supplementary independent expression estimates, and that the various miRNAs are potential biomarkers for different aspects of IBD.

Our main objective was to assess the differences in miRNA expression levels in rectal biopsies between pUC and aUC patients. Using RT-qPCR, we found that miR-21 was increased three-fold in aUC. miR-21 has repeatedly been reported to be increased in inflamed tissue in both pUC [31, 38, 39] and aUC [10, 13, 22] compared with healthy controls. Age related expression levels of miRNAs in IBD has, however, not previously been reported. It is not known if miR-21 levels in a normal colon are increased in adulthood, but systemic inflammation related to aging may impact on the miR-21 levels, as it has been reported for miR-155 [9]. Furthermore, the qISH analysis revealed a strong correlation between miR-21 and the severity of disease with increased expression estimates being associated with higher Geboes Score. High Geboes Score is based on the presence of inflammatory cells, crypt destruction and ulcerations [40]. Thus, miR-21 level in UC is an additional independent histological marker for disease severity when determined by qISH. Increased levels of miR-21 is common in cancer and in many types of non-neoplastic diseased tissues, such as atherosclerosis and psoriasis [41, 42]. miR-21 may be a general marker of disease, however, its mechanism of action is unknown [43] and may vary according to disease type. TNF- α is a potent pro-inflammatory cytokine elevated in IBD and used as a target in 2^nd^ line therapies [44]. Møller et al*.* [45] studied the potential interplay between the expression of miR-21 and TNF- α mRNA and reported a possible coordinated co-expression and suggested both paracrine and autocrine regulatory interplays.

Interestingly, DSS-induced colitis in miR-21 deficient mice was decreased compared to that in wild type mice and had reduced levels of TNF- α [21]. It is not known if anti-TNF- α therapy to IBD patients affects the levels of miR-21.

It is noteworthy that the miR-21 expression estimates obtained by RT-qPCR and qISH did not correlate, and neither were the significant correlation of miR-21 with disease parameters replicated with the two methods. In the IBD study by Thorlacius-Ussing et al*.* [10], miR-21 expression estimates from RT-qPCR and qISH obtained from FFPE biopsies showed weak but significant correlation. The lack of correlation in our study may be explained by technical differences, including use of small FFPE tissue biopsies causing compromised RNA recovery, use of an inadequate normalization approach in RT-qPCR and that qISH is performed in ROIs to include only intact diseased tissue. In addition, the RT-qPCR applied here measures only the mature miRNA [36], whereas qISH will detect both mature miR-21 and pre-cursor forms. Biogenesis of mature microRNAs is a multistep process that involves several enzymatic processing steps and can lead to variants in the mature form [46]. We therefore speculate that the lack of correlation between RT-qPCR and qISH is related to changes in processing of miR-21, which, together with the age-factor, could confound a direct correlation, implying that the increased levels measured by RT-qPCR in aUC compared to pUC could possibly be related to biogenesis of relatively more mature miR-21 in aUC.

Our data also showed that the miR-21 levels measured by RT-qPCR were higher in males compared to females. A similar sex-related difference was not found among the pediatric patients, however, similar sex-related miR-21 differences have been reported in CRC and hypertrophic heart disease [47–49]. Our findings support the assumption that miR-21 expression levels to some extent is regulated by hormone-related pathways.

QISH is rarely used to assess miRNA expression in UC, but the technique has a clear advantage over RT-qPCR including a visual inspection of the expression in the tissue and cellular compartments [10]. miRNA expression levels determined by qISH is a well-described method also used in other diseases and for other miRNAs [50, 51]. In this study, we found that RT-qPCR and qISH data provide independent expression level estimates that both show clinical relevance, as discussed above for miR-21. In our study we found miR-126 to be expressed in endothelial cells as expected and, thus, miR-126 levels represent a stage of vascularization. This was also found in the study by Thorlacius-Ussing et al. [10], who further found miR-126 expression to be increased in active IBD tissue compared to colon tissue from healthy controls. Therefore, the increased miR-126 expression likely reflects the increased vascularization in the IBD affected tissue [52], and further explains the strong correlation between miR-126 and both endoscopically and histologically assessed disease severity. A recent study reported that high miR-126 levels in endothelial cells inhibited inflammation in a mouse model of septic inflammation [53]. The increased vascularization in the inflamed tissue may therefore not only provide access for immune cells, but also contribute to protection against excess inflammation which could potentially be facilitated by miR-126 therapy [9].

MiR-142 was found to be negatively correlated with histologically assessed severity of disease in previously diagnosed patients, and therefore seems to be associated with an anti-inflammatory response in patients with longer duration of disease. The association with disease activity could potentially be explained by reduced levels of miR-142 in T-cells upon activation [34]. miR-142 is thought to target the ABCG2 and ABCB1 genes, which encode transport proteins and are important to the intestinal barrier regulation [54]. In a murine sepsis model, miR-142 was found to inhibit inflammation [55] and, moreover, miR-142 has been found to induce immune tolerance [56]. As UC is thought to develop, in part, due to loss of immune tolerance towards the gut microbiome leading to a breach of the intestinal barrier, this miRNA should be further evaluated in order to understand its possible anti-inflammatory effects and its role in IBD pathogenesis.

Both miR-31 and miR-155 were negatively correlated with the histological assessment of the disease and the strongest correlations were found in pUC (miR-155) and in previously diagnosed patients (miR-31), when stratifying according to patient characteristics. Moreover, the expression levels were significantly increased in patients in histologically assessed remission. In a study by Gwiggner et al*.*, miR-31 and miR-155 were both shown to target the IL-13 receptor α-1 mRNA [15]. While the effect of this is not fully understood, this receptor leads to increased phosphorylation of Janus Kinase in epithelial cells [57], which is the target of tofacitinib, a novel therapeutic drug shown to both induce and maintain remission in UC [58]. When combining our findings with the existing literature, both miR-31 and miR-155 seems to be promising candidates as therapeutic agents. The results from the study by Paraskevi et al. [20] reporting an increased expression of miR-155 in peripheral blood from UC patients, supports the rational for using anti-miR-155 as a therapeutic agent [59, 60].

The main strength of this study is the prospective inclusion of well-characterized patients of which half were newly diagnosed and therefore included before treatment was initiated. This allowed us to correlate the miRNA expression levels with clinical features, which is often lacking in the existing miRNA literature, and make the results from the present study more easily applicable to the problems faced in the daily clinical work with IBD patients. In conclusion, in prospectively collected FFPE rectal biopsies we found different expression levels and patterns of the examined miRNAs in pUC and aUC, which indicates differences in the etiology of pUC and aUC that might help explain the more severe disease course reported in pUC. The possible involvement of miRNA in the regulation of inflammation may compromise the efficacy of IBD therapies and calls for combined therapies directed towards partly overlapping pathways. Further studies on miRNA expression may help identify such novel relevant pathways and hopefully targets.

## MATERIALS AND METHODS

### Ethics Statement

All patients gave written consent before inclusion. If the patient was < 18 years old at time of inclusion, consent was given by both parents / guardians. The investigation has been conducted in accordance with the ethical standards and according to the Declaration of Helsinki and according to national and international guideline and has been approved by the National Ethical Committee (jr. nr.: H-16049534).

### Study population

In this prospective study, we obtained rectal biopsies from pediatric and adult patients scheduled for endoscopy (colonoscopy or sigmoidoscopy) due to either suspected UC or a flair of a previously diagnosed UC (diagnosed >one year prior to the present endoscopy). The biopsies were obtained from patients hospitalized from July 2017 to April 2018. Exclusion criteria were young age (< five years), previous cancer diagnosis, previous bowel resection and adult UC patients who were originally diagnosed < 18 years of age. Prior to endoscopy, patients symptoms were scored by the Pediatric Ulcerative Colitis Activity Index (PUCAI) and the Simple Clinical Colitis Activity Index (SCCAI) for pediatric and adult patients, respectively [61, 62]. At endoscopy, disease extent was recorded according to the Montreal classification [63] and severity was assessed by the Ulcerative Colitis Endoscopic Index of Severity (UCEIS [range: 0-8]) [64] and biopsies were instantly formalin fixed and subsequently embedded in paraffin for further diagnosis. The study was performed at the Department of Pediatrics and the Gastro Unit, Medical division, Copenhagen University Hospital, Hvidovre, Denmark.

### Histological assessment of the intestinal mucosa

The biopsies were formalin fixed and paraffin embedded and 3 μm sections stained with hematoxylin and eosin. The histological severity were graded as follows (the Geboes score [65]): 0, structural change only (grade 0-3); 1, chronic inflammation (grade 0-3); 2a, lamina propria eosinophils (grade 0-3); 2b, lamina propria neutrophils (grade 0-3); 3, neutrophils in epithelium (grade 0-3); 4, crypt destruction (grade 0-3); and 5, erosions or ulcers (grade 0-4). Histologic remission was defined as a Geboes score < 2. All assessments were made by the same IBD pathologist, who was blinded from the clinical presentation.

### MicroRNA RT-qPCR analyses

RNA Purification. Three paraffin sections of 5 μm thickness from each sample were placed in a 1.5 ml Eppendorf tube. RNA purification was performed using the miRNeasy FFPE Kit (Qiagen) according to the manufacturer’s instructions. In short, sections were deparaffinized in xylene and methanol, followed by permeabilization using Proteinase-K treatment and digestion with DNase to eliminate endogenous DNA fragments. RNeasy MinElute spin columns (Qiagen) were used for total RNA purification and finally eluted using RNase free-water. The RNA yield was determined using a NanoDrop 1000 (Thermo Scientific) and varied from 4 to 30 ng/μl.

For Reverse transcription quantitative polymerase chain reaction, we used MiRCURY LNA miRNA PCR assays for the determination of miRNA expression levels. Complementary DNA (cDNA) was obtained by reverse transcriptase (RT) incubation on 5 ng total RNA following the manufacturer’s instructions. For the miRCURY LNA SYBR Green PCR Kit we used primer pairs for hsa-miR-21-5p (miR-21), hsa-miR-31-5p (miR-31), hsa-miR-126-3p (miR-126), hsa-miR-142-5p (miR-142) and hsa-miR-155-5p (miR-155) (Qiagen). The RT-qPCR expression level (Ct value) of hsa-miR-103a-3p (miR-103) was used as endogenous reference miRNA based on previous observations [10]. RT-qPCR was run on Applied Biosystems QuantStudio Systems. Threshold and baseline settings were manually set and kept constant on all plates. All samples were run in duplicate and no-template controls were included. Melting curve analysis was performed in all runs to validate the PCR product.

### *In situ* hybridization and image analysis

Paraffin sections were cut at 5 μm thickness and placed on Super Frost Ultra Plus slides (Menzel Gläser). Slides were deparaffinized with xylene and rehydrated through decreasing concentrations of ethanol. Slides were placed on the Ventana Discovery Ultra Instrument (Roche) for automated hybridization [45]. In short, the tissue sections were digested with Proteinase-K. Double-digoxigenin-labelled probes for miR-21-5p (1 nM) and miR-126-3p (5 nM) were both hybridized at 60° C. Alkaline phosphatase-conjugated anti-DIG antibody was used for probe detection and followed by enzymatic color development with nitro blue tetrazolium (NBT-BCIP). Nuclei were counterstained with Nuclear Fast Red (Roche). Slides were removed from the instrument and dehydrated through increasing concentrations of ethanol. Finally, slides were mounted with Eukitt Mounting Medium. Two sections from each patient were stained as replicates. In some of the cases one or both sections were lost during the staining procedure. Digital whole slides were obtained using a 20X objective in a bright-field slide scanner (Axio Scan.Z1; Zeiss). To obtain relative expression estimates from the *in situ* hybridization analyses, we delineated a region of interest (ROI) of the total tissue area in each slide using image analysis software (Visiopharm, Hoersholm, Denmark) as also described previously [10]. The average ROI was 0.8 mm^2^. For each slide, we obtained area fractions of the blue ISH signal relative to the total tissue area, representing the expression level estimates of the quantitative image analysis of *in situ* hybridization (qISH). From duplicate sections we used the average of the two estimates in the statistical analyses. An overview of the image analysis process is presented in Supplementary Figure 1.

### Statistical analyses

Unless otherwise stated, descriptive data are presented as median and either interquartile range (IQR) or range for continuous variables and frequencies and percentages for categorical variables. RT-qPCR data was analyzed by the 2^–(∆Ct)^ method [66] using miR-103 as reference to yield relative fold expression levels of the different miRNA’s. The fold differences were compared between groups using the nonparametric Mann-Whitney U test. Spearman’s correlation coefficient was used to calculate the correlation between fold differences and continuous variables. P values were considered statistically significant when <0.05. Statistical analyses were performed using SAS Enterprise (version 7.11, SAS institute Inc., Cary. NC. USA).

## Supplementary Material

Supplementary Figures
