# Supplementary material for: Mucosal microRNAs relate to age and severity of disease in ulcerative colitis
Source: Aging (Albany NY). 2021 Mar 1;13(5):6359–74. doi: 10.18632/aging.202715 (PMC7993741; doi:10.18632/aging.202715)
Supplement: Supplementary Figures [file aging-13-202715-s001.pdf]

SUPPLEMENTARY FIGURES

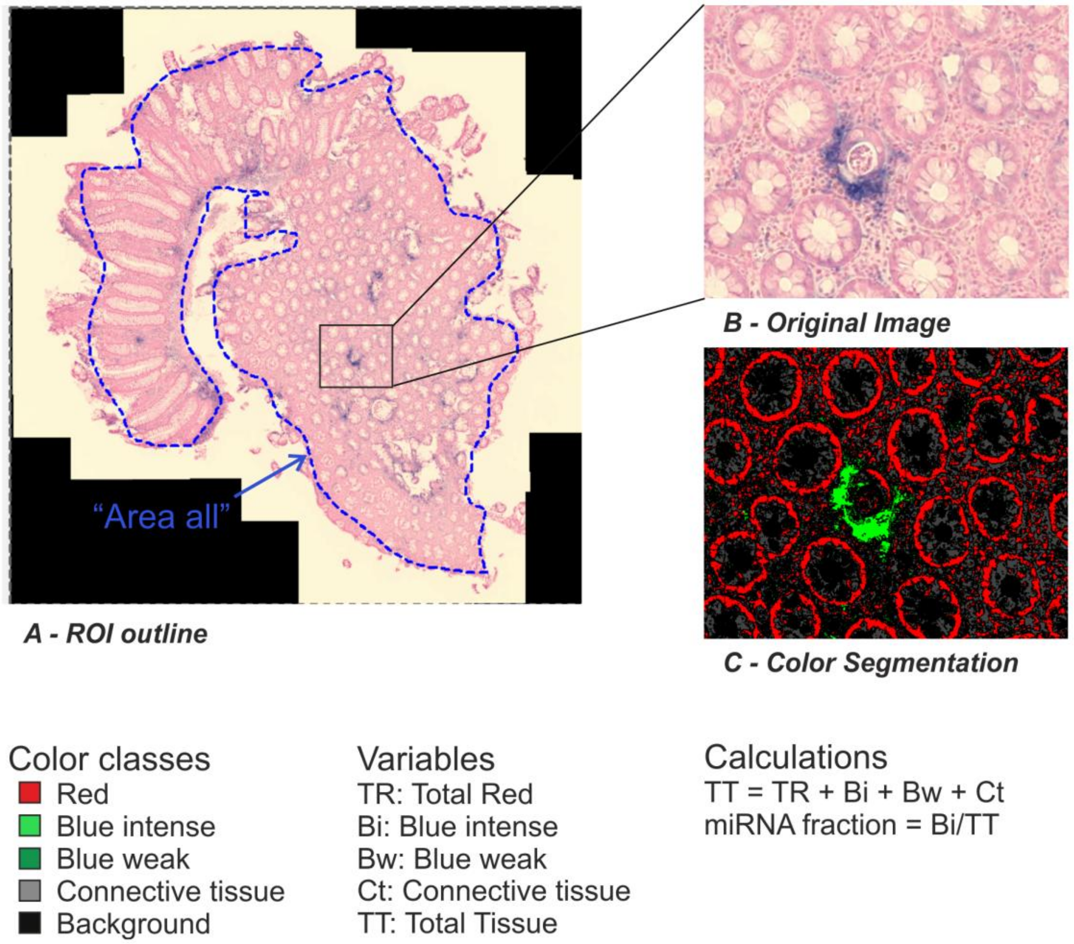

Supplementary Figure 1. Example of the image analysis for ISH using VisiomorphDP pixel classification of miR-21 stained section. ROI was selected manually for all the sections without any artefacts. miR-21 staining (blue) and Nuclear Fast Red background stain (red) in A and B. Pixel classification is shown in C. ISH, in situ hybridization. ROI, region of interest.

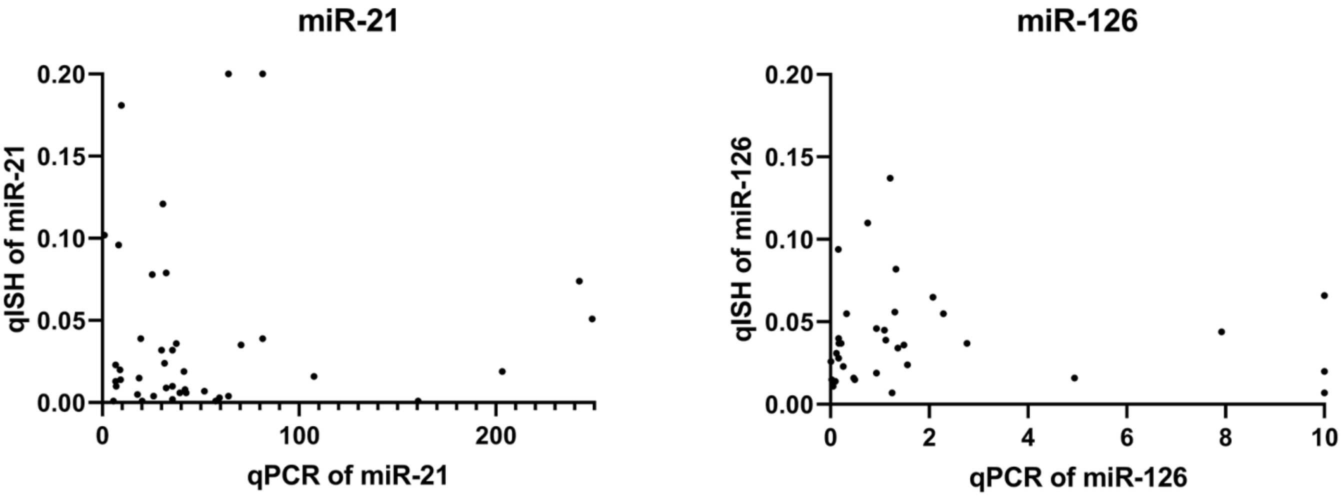

Supplementary Figure 2. The relationship between quantitative image analysis of situ hybridization (qISH) and reverse transcription quantitative polymerase chain reaction (RT-qPCR) quantification of miR-2 and miR-126 in the rectal mucosa of UC patients.
